# Optimizing THP-1 Macrophage Culture for an Immune-Responsive Human Intestinal Model

**DOI:** 10.3390/cells12101427

**Published:** 2023-05-19

**Authors:** Pornwipa Phuangbubpha, Sanya Thara, Patsawee Sriboonaied, Puretat Saetan, Wanwiwa Tumnoi, Adisri Charoenpanich

**Affiliations:** Department of Biology, Faculty of Science, Silpakorn University, Nakhon Pathom 73000, Thailand; phuangbubpha_p@silpakorn.edu (P.P.); thara_s2@silpakorn.edu (S.T.); sriboonaied_p@silpakorn.edu (P.S.); saetan_p2@silpakorn.edu (P.S.); tumnoi_w@silpakorn.edu (W.T.)

**Keywords:** THP-1, macrophages, immune response, inflammation, intestinal models, long term, cytokines, high cell density

## Abstract

Previously established immune-responsive co-culture models with macrophages have limitations due to the dedifferentiation of macrophages in long-term cultures. This study is the first report of a long-term (21-day) triple co-culture of THP-1 macrophages (THP-1m) with Caco-2 intestinal epithelial cells and HT-29-methotrexate (MTX) goblet cells. We demonstrated that high-density seeded THP-1 cells treated with 100 ng/mL phorbol 12-myristate 13-acetate for 48 h differentiated stably and could be cultured for up to 21 days. THP-1m were identified by their adherent morphology and lysosome expansion. In the triple co-culture immune-responsive model, cytokine secretions during lipopolysaccharide-induced inflammation were confirmed. Tumor necrosis factor-alpha and interleukin 6 levels were elevated in the inflamed state, reaching 824.7 ± 130.0 pg/mL and 609.7 ± 139.5 pg/mL, respectively. Intestinal membrane integrity was maintained with a transepithelial electrical resistance value of 336.4 ± 18.0 Ω·cm^2^. Overall, our findings suggest that THP-1m can be effectively employed in models of long-term immune responses in both normal and chronic inflammatory states of the intestinal epithelium, making them a valuable tool for future research on the association between the immune system and gut health.

## 1. Introduction

The responses of epithelial tissue to a pathogen or toxin involve a complex cascade of cellular interactions of defense mechanisms. Numerous *in vitro* co-culture models for studying the interaction of immune cells and other cell types, such as respiratory epithelium [1], intestinal epithelium, and cancer cells, have been proposed and employed [2]. Models of immune-responsive human intestinal epithelium are of interest because the intestinal epithelium is in direct contact with ingested contaminants and is an area of drug absorption, as well as the habitat of gut microbiota.

The Caco-2 human colon intestinal carcinoma cell line has been widely used for *in vitro* models of the human intestinal barrier [3]. Caco-2 cells spontaneously differentiate *in vitro* and display the characteristics of mature absorptive cells (enterocytes) with a microvilli brush border. Mucus-secreting cells, such as HT-29 and HT-29-methotrexate (MTX), can be incorporated into intestinal epithelium to increase its complexity [4]. Additional cytokines, such as interleukin (IL)-1β, tumor necrosis factor-alpha (TNF-α), interferon-gamma (IFN-γ), and lipopolysaccharides (LPS) are often utilized as inflammatory inducers for *in vitro* models of intestinal epithelial inflammation [2,5,6]. Immune cells are frequently incorporated into the culture since they facilitate interactions in both directions. However, primary immune cells, such as human peripheral blood mononuclear cell-derived monocytes, have a short life span of 20 h in peripheral blood and the inability to be expanded ex vivo [7]. Additionally, donor diversity results in considerable variability [7]. The utilization of immune cell lines for *in vitro* models has been proposed because they are homogenous, proliferate easily, and are simple to maintain in the laboratory. Furthermore, they can be obtained without contamination from other blood components. In contrast, the availability of primary human monocytes is limited [8].

The THP-1 cell line is derived from leukemia monocytes exhibiting a spherical single-cell suspension feature that can be stimulated to differentiate into macrophage cells. Macrophages play a crucial role in releasing pro- and anti-inflammatory cytokines in both acute and chronic inflammatory disorders [9]. Co-culture of Caco-2 cells with THP-1 macrophages (THP-1m) or conditioned medium from THP-1m culture has been utilized as a model of inflamed intestinal epithelium [5,10]. The interaction of macrophages and intestinal epithelium *in vitro* has been established [10,11]. It was reported that THP-1m-secreted cytokines, such as TNF-α, can promote cell death in intestinal epithelial Caco-2 monolayers [5]. Furthermore, Caco-2 nanoparticle absorption was enhanced with THP-1m co-culture [12].

There are many procedures that can be used to generate macrophages from THP-1, depending on the purpose of the investigation. The most common technique for THP-1m production is THP-1 stimulation with phorbol 12-myristate 13-acetate (PMA) at doses of 5–200 ng/mL for 24–72 h, followed by culture in new complete growth medium (CGM) for 2–5 days [13]. However, the percentage of detached THP-1m cells can reach up to 80.0% within 5 days after culture [14], with a high rate of apoptotic floating cells [15]. THP-1m co-culture with human alveolar epithelial cells extended the lifespan of co-cultured THP-1m up to 7 days, during which the epithelial cells maintained their tight barrier and the macrophages remained viable. However, a drop in the macrophage population may be observed [16]. Culture conditions significantly impact the development of THP-1m. High-density THP-1 cell cultures stimulate the expression of macrophage marker CD14 in response to PMA [17].

In this study, we examined and refined a 21-day culture of THP-1-derived macrophages and investigated its potential application in the creation of an immune-responsive intestinal epithelial model. THP-1m culture conditions, including cell density, PMA concentration, and media recipes were investigated. The macrophage features evaluated included cell morphology, cell adhesion, lysosome distribution, and cytokine secretions. Lastly, THP-1m was incorporated into the intestinal epithelium model with LPS-induced inflammation to demonstrate the inducible cytokine secretion of THP-1m in the immune-responsive intestinal model.

## 2. Materials and Methods

### 2.1. THP-1 Monocyte Cultures

The human leukemic cell line THP-1 was obtained from the Japanese Collection of Research Bioresources Cell Bank (Osaka, Japan; JCRB0112.1, lot#: 11102017). For cell proliferation, the cells were grown in T-25 flasks with an initial cell count of 2 million cells in a volume of 5 mL. The CGM was Roswell Park Memorial Institute 1640 (RPMI-1640; Gibco, Waltham, MA, USA; cat#: 21870076, lot#: 2051389) supplemented with 10% heat-inactivated fetal bovine serum (HyClone, Logan, UT, USA; cat#: SV30160.03, lot#: RB35957), 100 units/mL penicillin-streptomycin (HyClone; cat#: SV30010), and 2 mM L-glutamine (Corning Inc., Corning, NY, USA; cat#: SH30034.03, lot#: RB35957).

### 2.2. THP-1m Induction

To develop THP-1 into THP-1m for our long-term experiment, we seeded the cells at densities of 2 × 10^5^ and 1 × 10^6^ cells/well in six-well plates. PMA (Sigma-Aldrich, Saint Louis, MO, USA; cat#: 16561298) was evaluated at doses of 50, 100, and 200 ng/mL. After 48 h of PMA stimulation, THP-1m was obtained.

### 2.3. THP-1m Culture

After differentiation, all THP-1m cells were washed out of the PMA-containing medium and cultured in fresh CGM without PMA for 21 days. The medium was changed every 2 days. Two CGM recipes for long-term culture were used, namely, RPMI-1640 and Dulbecco Modified Eagle Medium (DMEM; HyClone; cat#: SH3002102) containing the same supplements of 10% heat-inactivated fetal bovine serum, 100 units/mL penicillin-streptomycin, and 2 mM L-glutamine.

### 2.4. THP-1m Morphology and Confluency

During the 21-day culture, changes in THP-1m morphology and confluency were observed. Microscopic images were taken on days 0, 5, 14, and 21 using a CK30 inverted microscope (OLYMPUS, Tokyo, Japan). Cell confluency was calculated as the percentage of cell-covered area. ImageJ software (National Institutes of Health, Bethesda, MD, USA) was used to analyze histogram function, and the black and white pixels were assigned for areas with and without cells, respectively. The percentage of cell confluency was determined by multiplying the ratio of the cell-covered area to the total area by 100.

### 2.5. THP-1m Detachment Rate

Cell adhesion is a widely accepted hallmark of the differentiation of monocytes into macrophages. During the culture of THP-1m, non-adherent cells were counted using a hemocytometer. The trypan blue (Fluka, Buchs, Switzerland; cat#: 093590) dye exclusion test was used to determine the number of detached dead cells present in a cell suspension.

### 2.6. Crystal Violet Staining

Crystal violet staining was used to detect the density of attached cells. Briefly, the cells were fixed for 10 min with 4% paraformaldehyde, washed three times with phosphate-buffered saline (PBS), and stained with 0.1% crystal violet (Fluka; cat#: 215298881) for 30 min. Thereafter, the samples were washed five times with PBS. Whole-well images were taken using a stereomicroscope. Absorbance were read at 540 nm (PACKARD, A153601, Palo Alto, CA, USA) [18].

### 2.7. LysoTracker and Nuclear Staining

LysoTracker red and Hoechst staining were used to analyze lysosome density and nuclear size and shape, respectively. The cells were washed twice with PBS and incubated in phenol red-free DMEM with 25 nM of LysoTracker red (Thermo Fisher Scientific, Waltham, MA, USA; cat#: L7528) for 30 min at 37 °C. Then the samples were washed twice with PBS and incubated with 5 µM of Hoechst stain (Abcam, Waltham, MA, USA; cat#: AB228551) for 30 min at 37 °C. Finally, the cells were washed twice with PBS. To determine the variation in lysosome density across distinct cell morphologies, the fluorescence area and intensity were measured using ImageJ software. The corrected total cell fluorescence (CTCF) was subsequently calculated as previously described [19]. The microscopic fluorescent images of the stained nuclei were analyzed with ImageJ using the particle analysis function to measure the maximum and minimum Feret diameter of the nuclei. The nucleus size was determined as the average of the maximum and minimum Feret diameter. The ratio of maximum to minimum Feret diameter was calculated to reveal differences in nuclear shapes.

### 2.8. Morphological Analysis

On days 5, 14, and 21, inverted microscopy was used to classify the type of THP-1 morphology and compare differences in cell morphology between the two different medium types. Macrophages were classified into two categories based on their morphology: round or spreading. The spreading category included “fried egg” and “spindle-like” morphologies [20]. The percentage of each cell type was calculated by counting a minimum of 300 cells from each experimental condition. The total number of cells per well was determined by multiplying the number of cells per unit area by the area of the well.

### 2.9. Culture of Intestinal Epithelial Cells

Caco-2 intestinal epithelial cells (cat#: HTB-37) and HT-29 cells (cat#: HTB-38) were purchased from the American Type Culture Collection (Manassas, VA, USA). The cells were seeded at a concentration of 5 × 10^5^ in T-75 flasks in DMEM-based CGM. The medium was changed every 2 days, and the cells were subcultured when they reached 80% confluency. To induce the differentiation of HT-29 cells into mucus-secreting goblet cells (HT-29-MTX), HT-29 cells were seeded at a concentration of 5 × 10^5^ in T-75 flasks for 5 days, incubated with 0.1 µM MTX (Sigma-Aldrich; cat#: SIA-M9929) for 28 days, and cultured in DMEM-based CGM without MTX for 48 h [21]. The medium was replaced every 2 days. After differentiation, HT-29-MTX cell lines were harvested and stored in liquid nitrogen until use. For cell proliferation investigations, the HT-29-MTX cells were cultured in CGM at a concentration of 5 × 10^5^ cells in T-75 flasks. After 4–5 days, 80% confluence was achieved.

### 2.10. Triple Co-Culture of Caco-2, HT-29-MTX, and THP-1m Cells

For the triple co-culture experiments, the *in vitro* intestinal model was cultured on double-chambered six-well plates with Costar Snapwell cell culture inserts (Corning; cat#: CLS3801-24EA, PET, 12 mm diameter, 0.4 µm pore size). Caco-2 cells (3.24 × 10^5^) and HT-29-MTX cells (3.6 × 10^4^) (ratio: 9:1) were cultured on the apical part of the insert with DMEM-based CGM. The media were changed every 2 days, with 3 mL of media in the basolateral part and 300 μL on the apical part. On day 18, THP-1 was cultured in parallel in the six-well plate to generate THP-1m. The cells were seeded at 1 × 10^6^ cells/well and incubated with 100 ng/mL PMA. After 48 h, they were washed out of the PMA-containing medium and given fresh medium without PMA for 24 h. On day 21 day, the culture inserts with Caco-2 and HT-29-MTX cells were transferred to the cell culture plate containing the THP-1m and cultured with DMEM-based CGM. Inflammation was induced by prolonged exposure to 100 ng/mL of LPS throughout the culture period of 21 days.

### 2.11. Scanning Electron Microscopy (SEM)

SEM was used to evaluate structures on cell surfaces, including mucus and microvilli. The samples were fixed with a 2.5% glutaraldehyde solution for 1 h, dehydrated through serial grades of ethanol (50%, 70%, 90%, and 100%) for 10 min each at room temperature, and then subjected to critical point drying (Quorum Technologies, Rewes, UK, K850) for 15–20 min. The membrane containing the cells was mounted on SEM specimen stubs. Then, the specimens were coated with gold/palladium and examined using a TESCAN MIRA3 SEM (TESCAN, Brno, Czech Republic).

### 2.12. Transepithelial Electrical Resistance (TEER) Assay

To determine cell monolayer integrity, TEER was evaluated using a Millicell ERS-2 Voltohmmeter (Merck, Darmstadt, Germany). The co-culture models were measured on days 3, 7, 14, and 21 before the experiment to monitor the development of the intact monolayer. Before the start of the experiment, TEER values > 300 Ω·cm^2^ were used for the *in vitro* intestinal model studies [22]. Then, the TEER values of the co-culture and triple co-culture models were measured in triplicate on days 3, 7, 14, and 21. The TEER values of the sample (Ω·cm^2^) were calculated by subtracting the TEER of the blank (filter insert alone) and then multiplying by the membrane area (cm^2^) [23].

### 2.13. Cytokine Secretion Measurement

The supernatant of Caco-2/HT-29-MTX co-culture and triple co-culture was collected following culture with DMEM-based CGM medium or 100 ng/mL of LPS. The TNF-α and IL-6 concentrations were determined using commercial TNF-α (Immunotools, Friesoythe, Germany; cat#: 31673019U1) and IL-6 (Immunotools; cat #31670069U1) enzyme-linked immunosorbent assay kits, as directed by the manufacturer. Briefly, 150 μL of the cell culture medium on the apical part and 1.5 mL of medium in the basolateral part were collected on days 7, 14, and 21. The samples were centrifuged at 1200 rpm for 5 min, and the supernatants were aliquoted and stored at −20 °C.

### 2.14. Lactate Dehydrogenase (LDH) Activity

An LDH activity assay (Sigma-Aldrich; cat#: MAK066) was used to measure LDH activity in accordance with the manufacturer’s instructions. Briefly, 50 μL of the supernatant, obtained in the same manner as for cytokine secretion measurement, was incubated with 50 μL of the master reaction mix for 3 min at room temperature. The LDH activity was determined by measuring the absorbance at 450 nm at 5-min intervals for 35 min. The activity (milliunits/mL) was then calculated by comparing the sample absorbance with the standard curve and reaction time using the following equation:

$$LDH\;activity=\frac{amount\;\left(nmol\right)\;of\;NADH\;generated\;between\;T\;initial\;and\;T\;final\;\times \;sample\;dilution\;factor}{\left(reaction\;time\right)\;\times \;sample\;volume}$$

where T = time.

### 2.15. Statistical Analysis

SPSS V25 statistical software (IBM Corp., Armonk, NY, USA) was used to analyze the data in this study. One-way ANOVA with Tukey multiple comparisons was used for equal variances and Dunnett C multiple comparisons for unequal variances. In cases of non-normal distribution, the Kruskal–Wallis test and Mann–Whitney U test were employed to establish the level of significance between the groups. The *p* values < 0.05 were considered significant. Data are presented as the mean ± standard deviation calculated from three experimental replicates unless otherwise specified.

## 3. Results

### 3.1. THP-1m Generation

**Figure 1:**
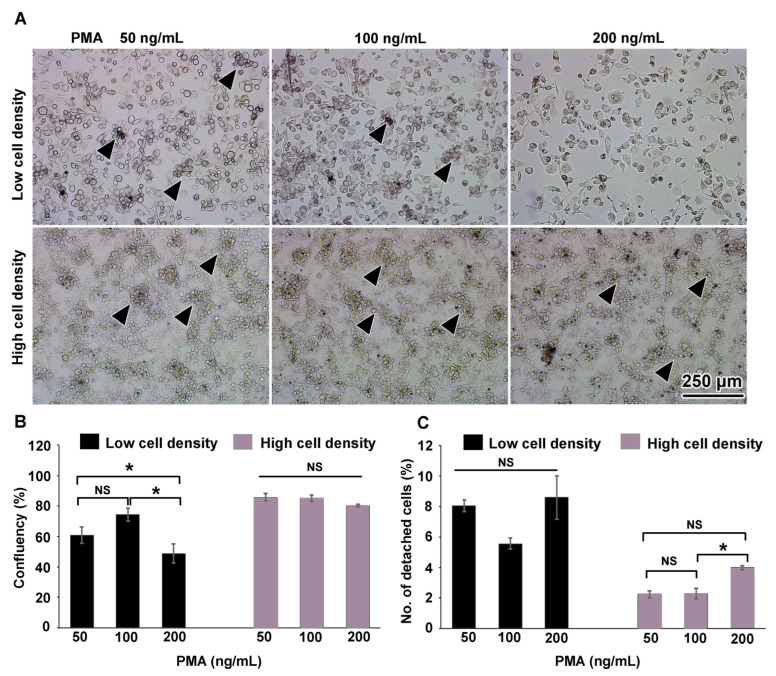
THP-1 macrophages on day 0. (**A**) The overall morphology of adherent cells and the round cell colonies linked with the adherent cells (arrowheads). (**B**) Percentage of area confluency analyzed with ImageJ. (**C**) Percentage of detached cells. Values are presented as mean ± standard deviation. Data were analyzed using ANOVA with Tukey test for multiple comparisons. * Statistically significant (*p* < 0.05). NS = not significant.

THP-1 monocytes were differentiated into THP-1m by stimulation with PMA at doses of 50, 100, and 200 ng/mL for 48 h. Two initial cell seeding densities were compared: a low density of 2 × 10^5^ cells and a high density of 1 × 10^6^ cells. All treatment conditions induced the differentiation of THP-1 to THP-1m, which were characterized by enlarged and adherent morphology (Figure 1A). At the high cell seeding density, large spherical colonies dispersed and interconnected with adherent cells were observed (Figure 1A). Colonies of non-adherent round cells also developed at the low cell seeding density with PMA concentrations of 50 ng/mL and 100 ng/mL, but to a lesser extent, and were not observed when the PMA concentration was 200 ng/mL (Figure 1A). Both cell seeding density and PMA concentration affected the number of adherent THP-1m cells (Figure 1A). An image analysis program was used to evaluate cell confluency. At the low cell seeding density, THP-1m cells induced by PMA at 50 and 100 ng/mL showed a confluence of 60.8% ± 5.2% and 74.3% ± 4.1%, respectively, which was significantly greater than the cells induced by 200 ng/mL PMA (48.9% ± 6.3%; *p* < 0.05). At the high cell seeding density, the average percentage of confluency was 83.7% ± 2.5%, which was significantly greater than the average percentage of confluency at the low cell seeding density (61.3% ± 10.4%; *p* < 0.05) (Figure 1B). Since cell shape and colony formation substantially impact the number of cells per surface area, an equal percentage confluency may not directly correspond to an equal number of cells. The detachment rates were calculated by comparing the number of detached cells with the initial number of cells. At the low cell seeding density and PMA concentrations of 50–200 ng/mL, THP-1m cells had a detachment rate of 7.4% ± 1.3%. At the high cell seeding density, THP-1m had a lower detachment rate of 2.8% ± 0.8% (*p* < 0.05) (Figure 1C). Additionally, at the high cell seeding density, 200 ng/mL PMA significantly increased the rate of cell detachment compared with 100 ng/mL PMA (*p* < 0.05) (Figure 1C).

### 3.2. Long-Term (21-Day) Culture of THP-1m

**Figure 2:**
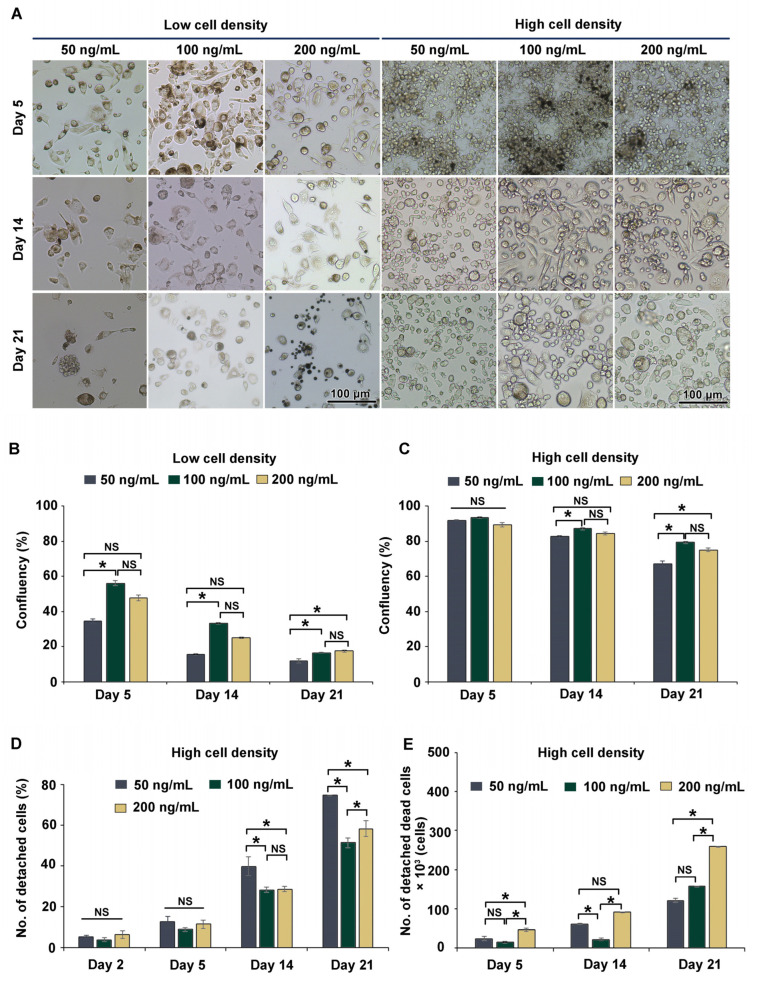
Long-term (21-day) culture of THP-1m. (**A**) Microscopic images showing cell morphology during the long-term culture. (**B**) Percentage of cell confluency of THP-1m from the low cell seeding density. (**C**) Percentage of cell confluency of THP-1m from the high cell seeding density. (**D**) Percentage of detached cells and (**E**) number of detached dead cells in THP-1m from the high cell seeding density. Values are presented as mean ± standard deviation. * Statistically significant (*p* < 0.05). NS = not significant.

The process of THP-1m differentiation is transient and reversible [14]. To observe morphological alterations and cell confluency in a long-term culture, THP-1m cells obtained after 48 h PMA induction were cultured for 21 days. As expected, there was a dramatic loss of adherent cells, especially those seeded at the low cell density (Figure 2A). Enlarged and spindle cells were observed in the cells seeded at the high cell density throughout the 21 days of culture (Figure 2A). The percentage of confluency was 15.2% ± 2.5% on day 21 in cells seeded at the low cell density and 73.8% ± 5.1% at the high cell density. On day 21, THP-1m induced by PMA at 100 and 200 ng/mL showed a significantly higher percentage of confluency compared with PMA at 50 ng/mL for both cell seeding densities (Figure 2B,C). However, many dead cells appeared as multiple dark spots in THP-1m induced by 200 ng/mL PMA (Figure 2A).

Further investigations were performed to examine the number of detached cells and the extent of cell death of THP-1m at the high cell seeding density. The trypan blue exclusion assay was used to count the detached dead cells. PMA at 200 ng/mL significantly increased the number of dead cells, corroborating the cell confluency results (Figure 2D,E). Therefore, THP-1m seeded at the high cell density and induced by PMA at 100 ng/mL was selected for the subsequent experiments comparing cell confluency, number of detached cells, and number of detached dead cells.

### 3.3. DMEM Promoted the Long-Term Maintenance of THP-1m Cultures

**Figure 3:**
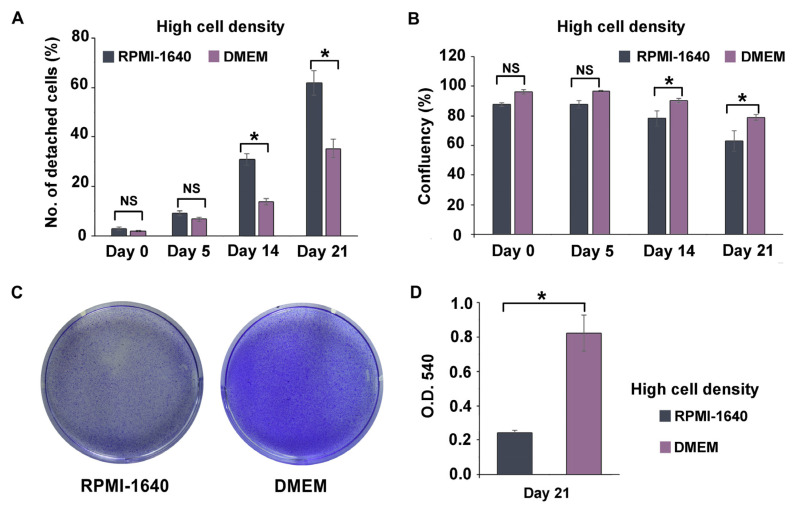
Long-term culture of THP-1m in RPMI-1640 and DMEM. (**A**) Percentage of detached cells. (**B**) Percentage of cell confluency. (**C**) Representative image of crystal violet staining on day 21. (**D**) Quantitative results of crystal violet staining on day 21. Values are presented as mean ± standard deviation. * Statistically significant (*p* < 0.05). NS = not significant.

The method of high cell seeding density in combination with PMA at 100 ng/mL for 48 h was selected based on prior results and preliminary findings (Supplementary Materials Figure S1). RPMI-1640 is a typical culture medium for non-adherent cells, whereas DMEM is used for adherent cells, including intestinal epithelial cell lines. DMEM is typically used if only one type of media is selected for co-culturing macrophage cells with other epithelial cells. Further analysis was conducted on the two media recipes for the long-term culture of THP-1m to confirm whether RPMI-1640 is required in the co-culture system. Interestingly, the results showed that THP-1m culture in DMEM helped in maintaining its 21-day culture. Similar cell morphology of enlarged and elongated THP-1m was observed in both culture media (Figure S2A). During the first 5 days, the number of detached cells and the percentage of cell confluency did not differ significantly. However, after 14 days, THP-1m cells cultured in RPMI-1640 showed a significantly higher percentage of detachment (17.2–26.5%) than those cultured in DMEM, and DMEM outperformed RPMI-1640 by approximately 11.9%–15.8% in terms of increased cell confluency (Figure 3A,B). Crystal violet staining was also conducted on day 21, confirming that DMEM significantly increased the number of THP-1m in long-term culture compared with RPMI-1640 (Figure 3C,D and Figure S2B).

**Figure 4:**
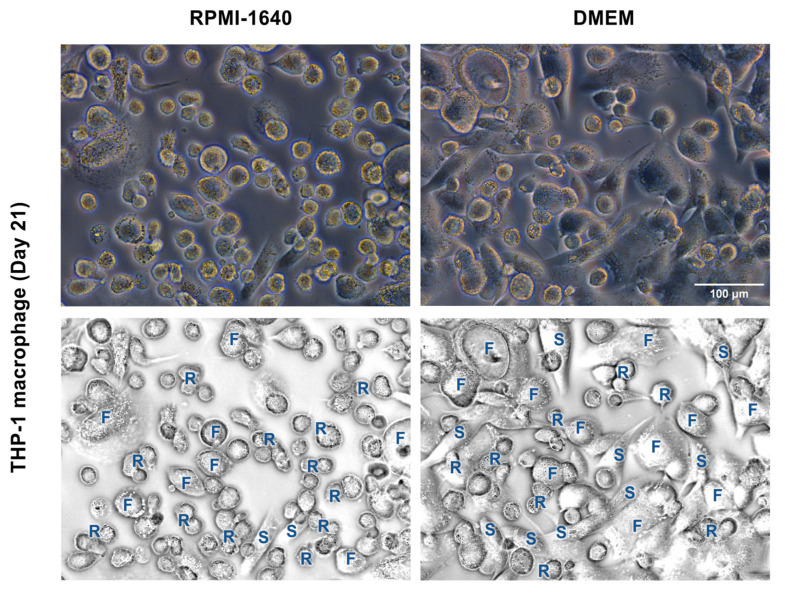
Morphology of THP-1m after 21 days in RPMI-1640 and DMEM. Top panels show the phase-contrast microscopic images. Bottom panels show examples of identified morphologies. R = round, S = spindle-like, and F = fried egg.

Additionally, changes in THP-1m morphology were monitored during the course of the 21-day culture. Three primary macrophage morphologies were identified: round cells, spindle-like cells, and fried egg cells (Figure 4). Observation of the THP-1m morphology revealed that DMEM not only aided THP-1m adherence but also significantly enhanced the proportion of THP-1m spindle-like cells and reduced the proportion of round cells and fried egg cells (Figure 4 and Table 1).

**Figure 5:**
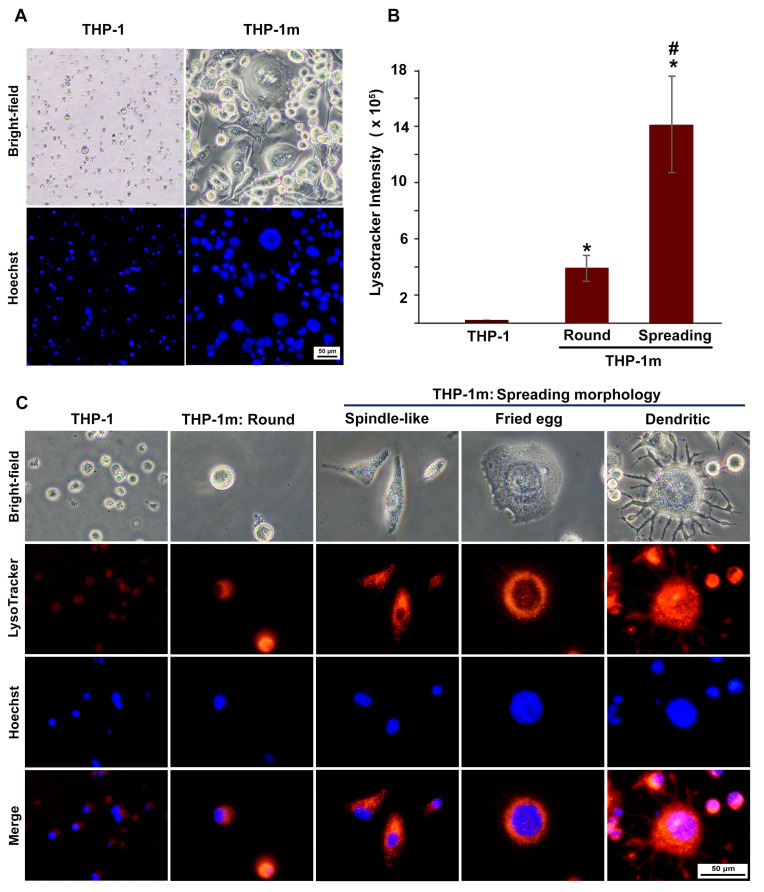
THP-1 macrophage (THP-1m) morphologies: increasing cell and nucleus size and expanding lysosomes. (**A**) Morphological comparison between THP-1 and THP-1m. Bright-field microscopy and nuclei staining with Hoechst dye showed changes in THP-1m morphology with increased cell and nucleus sizes. (**B**) Quantitative results of lysosome density shown as CTCF values (mean ± standard deviation). * *p* < 0.05 compared with THP-1 monocyte, # *p* < 0.05 compared with THP-1m (round). (**C**) Co-staining with Hoechst and LysoTracker revealed distinct THP-1m morphologies and an increased number of lysosomes.

It was shown that THP-1 monocytes had a fairly uniform morphology, with a size of 10.6 ± 1.5 µm and the ratio closest to 1.0, and not all THP-1 monocytes could be stained with Hoechst (Figure 5A and Figure S3). The nuclei of rounded THP-1m were twice as large as those of THP-1 monocytes, and they were four to five times larger in spindle-shaped and fried egg cells, respectively (Figure 5A and Table S1).

Lysosome expansion was confirmed in all of the THP-1m cell morphologies (Figure 5B,C). A significant increase in lysosome density was observed in THP-1m cells with spreading morphology compared with round cells. This increase was more than 4-fold, as indicated by the CTCF value (Figure 5B). A small number of fried egg cells displayed dendritic morphology, particularly at later time points (Figure 5C).

### 3.4. Inducible Cytokine Secretions While Maintaining Membrane Integrity in Long-Term (21-Day) Triple Co-Culture of THP-1m with Intestinal Epithelium Caco-2/HT-29-MTX

**Figure 6:**
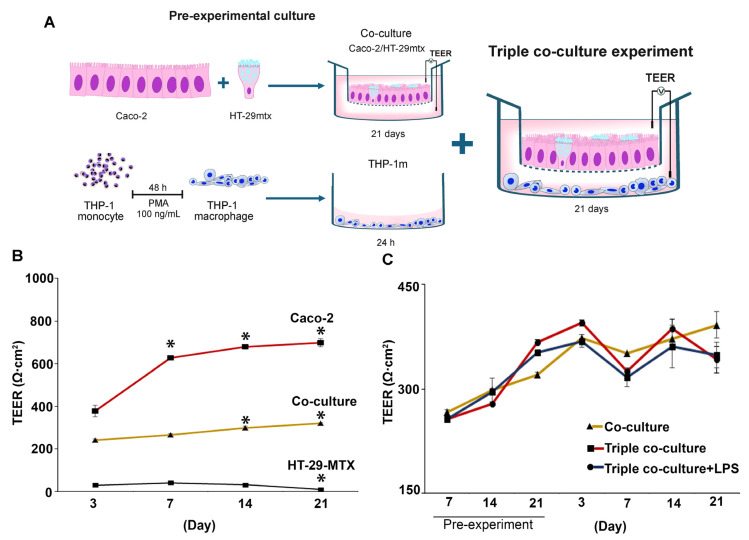
Changes in transepithelial electrical resistance (TEER) in triple co-culture of THP-1m with intestinal epithelium. (**A**) Schematic illustration of the triple co-culture setup. (**B**) TEER values were measured in monoculture and co-culture before the experiment, and (**C**) in the triple co-culture for a 21-day period. Values are presented as mean ± standard deviation. * Statistically significant compared with day 3 of the same culture (*p* < 0.05).

Co-culture of Caco-2 with HT-29-MTX at a ratio of 9:1 was performed 21 days prior to adding the THP-1m to the culture (Figure 6A). As expected, HT-29-MTX addition to the intestinal epithelium reduced the TEER. However, TEER increased over time and reached a minimum of 250 Ω·cm^2^ on day 7 (Figure 6B). After 21 days of co-culture, the TEER value reached 320.8 ± 18.9 Ω·cm^2^.

TEER analysis showed that the membrane integrity was maintained, with the average TEER values > 300 Ω·cm^2^ in the triple co-culture (358.7 ± 33.5 Ω·cm^2^) and the triple co-culture plus LPS (336.4 ± 18.0 Ω·cm^2^) compared with 367.5 ± 16.4 Ω·cm^2^ for the co-culture (Figure 6C).

**Figure 7:**
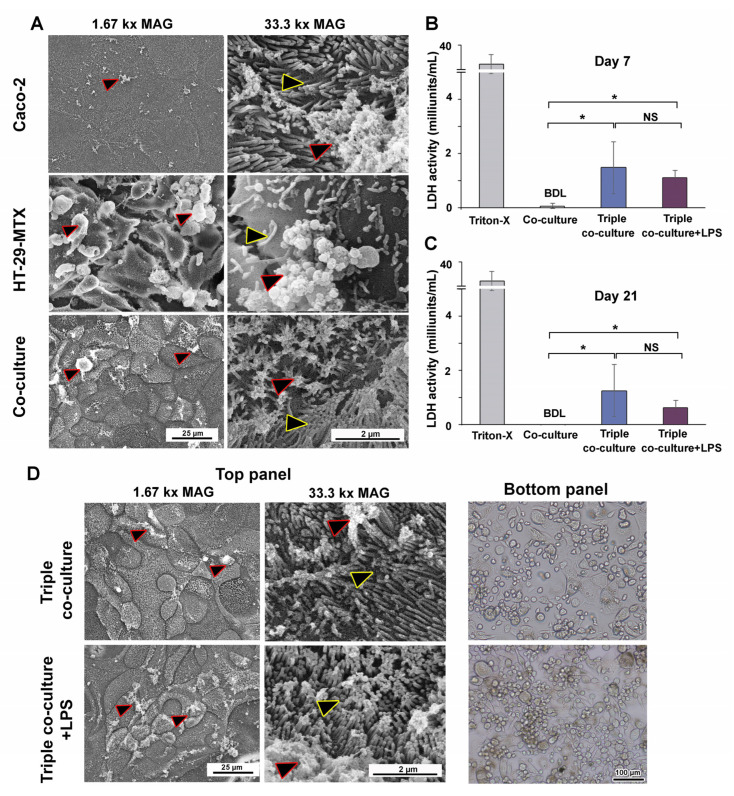
Morphological observation of intestinal epithelium and THP-1m in triple co-culture and the release of LDH in the supernatant. (**A**) SEM images of the apical surface of cultured intestinal epithelium (yellow outlined arrowheads: microvilli, red outlined arrowheads: mucus). (**B**,**C**) LDH release into the supernatants of the co-culture, triple co-culture, and triple co-culture with LPS-induced inflammation on days 7 and 21. Values are shown as mean ± standard deviation. BDL = below the detection limit. * Statistically significant (*p* < 0.05). NS = not significant. (**D**) SEM images of the apical surface of the cultured intestinal epithelium and cell morphology of THP-1m in triple co-culture and triple co-culture with LPS-induced inflammation after 7 and 21 days.

SEM was used to observe the apical surface of the intestinal epithelium. Caco-2 appeared well-organized, with evenly dispersed microvilli and modest mucus secretion (Figure 7A). HT-29-MTX appeared to be a disorganized cell layer with dispersed microvilli and high amounts of mucus (Figure 7A and Figure S4). The addition of HT-29-MTX enhanced the amount of mucus in the culture without compromising the organization of the single-layer polarized intestinal epithelium (Figure S4 and Figure 7A).

The LDH cytotoxicity assay results indicated that LDH release by Caco-2/HT-29-MTX co-culture was below the detection limit (Figure 7B,C). LDH was detected at low levels in the triple co-culture model, with a minimal release of 1.5 ± 1.0 milliunits/mL on day 7 and 1.1 ± 0.3 milliunits/mL on day 21. LPS addition did not increase LDH release, and the levels remained within 0.6–1.3 milliunits/mL (Figure 7B,C). SEM images revealed intact polarized intestinal epithelium in the top panel of the triple co-culture with and without LPS, while bright-field images of the bottom panel revealed the adherent THP-1m cells (Figure 7D).

**Figure 8:**
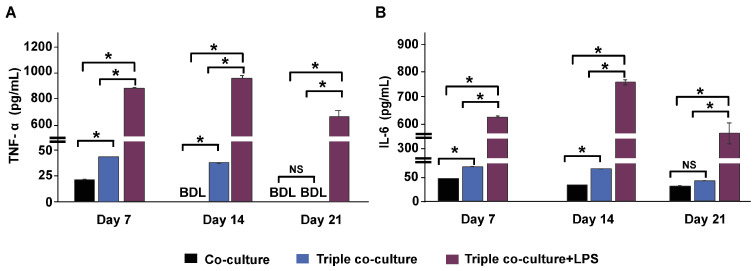
Cytokine secretions in triple co-culture and triple co-culture with LPS-induced inflammation. The levels of tumor necrosis factor-alpha (TNF-α) (**A**) and interleukin (IL)-6 (**B**) in culture media were measured on days 7, 14, and 21 during a 21-day long-term culture by collecting half of the culture media from both the apical panel and basolateral panel. Values are presented as mean ± standard deviation. BDL = below the detection limit (TNF-α: 22 pg/mL, IL-6: 6 pg/mL). * Statistically significant (*p* < 0.05). NS = not significant.

The levels of two secreted cytokines, TNF-α and IL-6, were analyzed from the culture media. An increase in TNF-α and IL-6 by adding THP-1m to the culture was observed, especially during the first 14 days of triple co-culture (Figure 8A,B). Without THP-1m, the level of secreted TNF-α was 23.0 ± 1.6 pg/mL during the first 7 days and was below the detection limit (22 pg/mL) (Figure 8A). The addition of THP-1m to the culture system resulted in a 1.76-fold increase in TNF-α levels (40.4 ± 1.2 pg/mL) on day 7 (Figure 8A). Inflammation induced by LPS significantly raised the amount of TNF-α released to 934.0 ± 25.8 pg/mL on day 14 and 642.0 ± 57.9 pg/mL on day 21 (Figure 7A). Without LPS induction, IL-6 was secreted at a higher level than TNF-α, with an average level in triple co-culture of 64.6 ± 14.3 pg/mL. LPS stimulation increased the level of IL-6 to 625.2 ± 6.9 pg/mL, 772.4 ± 19.1 pg/mL, and 431.6 ± 75.8 pg/mL on days 7, 14, and 21, respectively (Figure 8B).

## 4. Discussion

Macrophages are crucial inflammatory effectors that play essential roles in pathogen identification, inactivation of foreign molecules, protection against infection, wound healing induction, and cytokine production, which regulates the stages of inflammation [24,25,26]. Because they communicate and regulate the internal milieu of tissues, they are both the cause and treatment target of many diseases [27,28]. However, macrophage culture remains challenging, particularly for long-term investigations. In this study, the protocol for THP-1-derived macrophages was investigated and optimized, along with its application in a co-culture intestinal model in a long-term 21-day experiment.

THP-1 has been extensively used as a macrophage model *in vitro*. A study comparing PMA-induced THP-1m with primary cells of human monocyte-derived macrophages (MDM) reported THP-1m as a suitable alternative for assessing bacterial absorption and host response to drug-susceptible and drug-resistant mycobacterial infections [29,30]. Additionally, a study comparing the host responses of primary and secondary MDM cell lines and THP-1-like macrophages to pathogens did not find any significant differences in both pro-inflammatory cytokines and chemokines between the two cell types at 24 and 96 h post-infection [30]. These features make THP-1 an attractive model for researchers studying macrophages and their role in the immune response.

Various chemical treatments and culture methods have been proposed in an attempt to establish a protocol for THP-1m culture. A common protocol for THP-1m production involves treating THP-1 cells for 48–72 h with PMA at concentrations of 5–200 ng/mL [14,31,32,33,34,35]. Low concentrations of PMA (<5 ng/mL) were suggested as the optimal technique for studying the responses of THP-1m to weak stimuli in a short-term 24-h experiment [33]. However, THP-1m derived from PMA stimulation at concentrations of 5–15 ng/mL detached within 5 days of culture, but the adherence of THP-1m derived from PMA stimulation at concentrations of 30–125 ng/mL remained >20% after 5 days [14]. Moreover, 100–200 ng/mL PMA promotes macrophage differentiation, resulting in higher expression levels of the macrophage surface marker CD11b than with 25 ng/mL PMA [36]. Our preliminary results confirmed that PMA doses of 50–200 ng/mL can be used to create THP-1m cells for short-term experiments. However, after prolonged culture (14–21 days), most of the cells detached, and the remaining adherent cells lost their characteristic macrophage morphology. Besides PMA concentration, this study identified and evaluated other variables for their potential influence on the production of THP-1m cells and the preservation of macrophage morphology over time.

While searching for the optimal THP-1m culture procedure, we discovered that various cell concentrations, ranging from 1 × 10^5^ to 2 × 10^6^ cells/mL, had been utilized [14,29,36]. Since it was shown that a high cell seeding density could improve THP-1m characteristics by increasing CD14 expression [17], cell density was also compared in this study. We found that increasing the initial number of seeded cells significantly reduced the rate of detachment and affected cell morphology. When THP-1m cells were derived from PMA at a concentration of 100 ng/mL and seeded at a density of 1 × 10^6^ cells/well in a six-well plate, the detachment rate was <60.0% after 21 days of culture, and the cells displayed enlarged and elongated shapes. In contrast, when the cell density was reduced to 2 × 10^5^ cells/well, the detachment rate was >90.0%, and the cells lost their enlarged and elongated shapes. Macrophages cluster together to communicate with each other through secreted chemokines and cytokines and through actin-based tunneling nanotubes [37]. It was suggested that intercellular communication may play a role in the maintenance of macrophage populations, but further research is necessary to fully understand this process. Thus, the use of THP-1m as a model system could provide valuable insights into these mechanisms.

The standard media recipe for culture of suspension cells, such as THP-1, is RPMI-1640-based medium. However, DMEM-based media is preferred when co-culturing with epithelial cells. Since THP-1m are adhesion cells, changing their culture medium to DMEM may have a beneficial effect on their maintenance. We found that the use of DMEM reduced the rate of THP-1m cell detachment in a long-term 21-day culture. Additionally, DMEM enhanced the proportion of THP-1m spindle-like cells and reduced the proportion of round cells compared with RPMI-1640. The primary difference between these two media is that DMEM has a higher calcium concentration and a lower phosphate concentration than RPMI-1640. Calcium signaling is essential for cytoskeleton organization and the function of integrins, which are cell surface proteins responsible for cell-to-cell communication and cell adhesion to the extracellular matrix [38]. Previous research on mouse macrophages demonstrated that an intracellular calcium antagonist decreased PMA-induced cell spreading, whereas the addition of calcium ionophores increased PMA-induced cell spreading by 30% [39]. In mouse bone marrow-derived macrophages, calcium ion influx also played a critical role in the activation and formation of the M1 inflammatory phenotype [40]. We have shown here that the calcium ion concentration in the media may affect macrophage culture, and further investigation is needed to optimize its use.

In this study, the utilization of THP-1m as immune-responsive cells in a long-term co-culture model was established. THP-1m cells were triple co-cultured with Caco2 human intestinal epithelial cells and HT-29-MTX in a normal and inflamed state. In the triple co-culture system, low levels of cytokine secretions were detectable for up to 14 days for TNF-α (<34 pg/mL) and 21 days for IL-6 (<44 pg/mL). The reported cytokine levels of triple co-culture were within the ranges of TNF-α (44 ± 1 0 pg/mL) and IL-6 (27 ± 4 pg/mL) secreted by THP-1m after 24 h of culture [41]. A previous study showed that IFN-γ and IL-6 stimulate IL-6 secretion in isolated colonic crypts of mice from both epithelial and non-epithelial cells in the colonic mucosa [42]. Minimal cytokine secretion (<22 pg/mL of TNF-α and <43 pg/mL of IL-6) was observed in epithelial co-culture without THP-1m. This confirmed the significance of communication from epithelial cells, which may also affect macrophage responses [43]. No pro-inflammatory cytokines (IL-1ꞵ, IL-8, or TNF-α) were found in Caco-2/HT-29-MTX co-cultures and Caco-2 monocultures [2,11]. However, this may have been affected by IL-1ꞵ, low cell density, and detection being performed too early during the course of the experiment. Basolateral stimulation with IFN-γ can induce apical secretion of IL-6 in Caco-2 cells, leading to autocrine activation of STAT3 in intestinal epithelial cells [42]. In this study, we measured cytokine levels obtained from a combination of media samples collected from both apical and basolateral secretions. However, we suggest that future studies compare cytokine secretion among different panels because the membrane integrity of the epithelial barrier may cause compartmentalized responses.

Long-term exposure to LPS in the triple co-culture model led to the continuous upregulation of TNF-α and IL-6, which peaked on day 14, followed by a subsequent drop on day 21. Short-term experiments also demonstrated temporal expression of cytokine secretion during LPS induction. In a co-culture of lung epithelial cells with THP-1m, TNF-α and IL-6 levels were measured at 6, 12, 24, and 48 h, with peak expression observed at 24 h and a substantial drop at 48 h during the 48-h LPS exposure period [44]. In another short-term 48-h study, the transient cytokine secretion was pre-screened in IFN-γ pre-activated THP-1 cells following continuous exposure to 1 mg/mL LPS. The peak expression of TNF-α occurred at 3 h, followed by a drop after 6 h, while the peak expression of IL-6 was observed between 24 and 48 h [45]. Pre-exposure of macrophages to LPS is widely used in short-term experiments, resulting in the temporal expression of cytokines during the experimental period [46]. To conduct long-term studies, it is important to determine the appropriate concentration and duration of LPS exposure according to the research objectives. This varies depending on the specific experiment being conducted.

The levels of TNF-α and IL-6 substantially increased in LPS-induced inflammation, ranging from 642.0 ± 57.9 to 934.0 ± 25.8 pg/mL and from 431.6 ± 75.8 to 772.4 ± 19.1 pg/mL, respectively, measured on days 7, 14, and 21. In an inflamed intestine model of Caco-2 co-cultured with THP-1, exposure to IFN-γ and LPS caused TNF-α production (870.0 pg/mL) after 4 h of co-culture [2]. The up-regulation of TNF-α and IL-6 varies depending on the LPS concentration, cell types, cell numbers, and the experimental time points. In a short-term co-culture experiment lasting <48 h, Caco-2/HT-29-MTX cells were co-cultured with immune cells, including THP-1m, RajiB, and monocyte-derived dendritic cells, resulting in TNF-α levels of 900–2200 pg/mL [11,47,48] and IL-6 levels of 300–1200 pg/mL [49]. In monocultures of THP-1m or RajiB cells treated with LPS, the secretion levels of TNF-α and IL-6 were 200–2200 pg/mL and 100–900 pg/mL, respectively [14,29,49,50]. An increase in cytokine secretion in monocultures of Caco-2 upon LPS induction has been reported, with TNF-α levels of 60–140 pg/mL [51,52].

While cytokine secretion was significantly increased in LPS-induced inflammation in this study, TEER values were maintained >300 Ω·cm^2^. Achieving a balance among cell density, the ratio of Caco-2/HT-29-MTX, and prolonged culture may improve membrane integrity of the intestinal epithelium [22,53,54]. Additionally, mucus production from HT-29 and HT-29-MTX could help to protect membrane integrity [55]. LPS was reported to increase mucus secretion and the expression of the tight-junction component gene *ZO1* in a Caco-2/HT-29 co-culture. This mucus layer helps to prevent LPS from damaging the membrane. Only when the mucus layer was not intact, due to the silencing of *MUC2*, was LPS found to damage the tight junctions of the co-cultured cells [56]. Additionally, LPS concentrations ≤100 ng/mL did not cause apoptosis or affect the proliferation of Caco-2 cells [57]. Consistent with the findings of this study, minimal LDH release was observed in our triple co-culture model. Moreover, no significant changes were observed in the morphology of the intestinal epithelium or in terms of increased cell death following LPS treatment over the 21-day long-term culture period. Another factor that could interfere with TEER values is cellular swelling because it was reported that amine-modified polystyrene nanobeads increased TEER values by reducing intercellular spaces resulting from low-level necrotic events [58]. It was reported that membrane integrity could recover after damage, and a reduction in TEER by 10 ng/mL of LPS was observed within 4 h, but the value returned to normal within 48 h [2]. In this study, the inflammatory model was prolonged for 21 days with TEER measurement intervals of 3–7 days, so recovery of membrane integrity could be expected. Further investigation is needed to fully understand the factors affecting membrane integrity and TEER values in the intestinal epithelium. Adjustments in LPS treatments, variations in cell culture conditions, and additional measurements of membrane integrity, such as transepithelial flux and electrical cell-substrate impedance sensing, could provide more insights into the recovery of membrane integrity.

Intestinal macrophages play critical roles in regulating pro-inflammatory cytokine secretion, which is essential for controlling inflammatory responses in the intestinal wall. However, it is important to note that the immune responses in the intestinal wall are complex and involve the interaction of multiple immune cell types. The lamina propria of the intestinal wall contains both innate and adaptive immune cells, such as macrophages, dendritic cells, mast cells, T-cells, B-cells, and innate lymphoid cells [59]. Crosstalk between these immune cells is crucial for maintaining immune homeostasis in the gut. The crosstalk between gut macrophages and T-cells also regulates T-cell expansion and differentiation [60]. This study presents a protocol for long-term THP-1m culture and the utilization of THP-1m as immune-responsive cells in a triple co-culture intestinal epithelium model with inducible cytokine secretion. However, to better understand the complexity of the gut immune response, further studies using other immune cells are necessary.

## 5. Conclusions

The co-culture of intestinal epithelium and immune cells has proven valuable for gut inflammation research, drug testing for inflammatory bowel diseases, and understanding the interaction of food in the gut microenvironment [50,61,62]. However, these studies tend to be of a limited duration due to the dedifferentiation of macrophages that occurs during *in vitro* culture. This study provided a useful protocol for culturing THP-1m and demonstrated their potential for use in long-term macrophage-immune-responsive co-culture models. By using an adequate amount of PMA for induction and a high initial cell seeding density, THP-1m cells with inducible cytokine secretion capacity can be co-cultured with an intestinal epithelium model for up to 21 days to create both normal and chronic inflammatory intestinal epithelium models. Although this study focused on the application of THP-1m for long-term co-culture with intestinal epithelium, the protocols for obtaining THP-1m that can be cultured for 21 days are not limited to the triple co-culture intestinal model. Other models that incorporate immune cells could also benefit from adjusted protocols, including the recently developed intestinal *in vitro* model of mini-gut or gut-on-a-chip [63], as well as the microwell-based intestinal organoid-macrophage co-culture system [64]. Other existing models that incorporate immune cells include liver organoids, tumor-on-a-chip, immune-system-on-a-chip, and liver fibrosis models [65,66,67]. Overall, these adjusted protocols may enhance the utility of *in vitro* co-culture models for studying the immune response in various disease states.

## Figures and Tables

**Table 1:** Changes in THP-1m cell morphology following 21 days of growth in RPMI-1640- and DMEM-based CGM. The percentage of different cell morphologies (round cells, fried egg cells, and spindle-shaped cells) and the total number of cells are shown. Data are presented as average ± standard deviation.

Class					
	Day	Round (%)	Fried Egg (%)	Spindle-like (%)	Total Cell Number/Well
RPMI-1640	5	84.0 ± 4.1	1.8 ± 0.6	14.2 ± 2.4	4396.7 ± 1516.8
	14	65.2 ± 2.1	11.0 ± 1.4	23.8 ± 1.4	2210.0 ± 545.2
	21	82.1 ± 21.7	15.7 ± 3.1	2.2 ± 0.6	1835.0 ± 615.0
DMEM	5	63.2 ± 1.4	0.5 ± 0.1	36.3 ± 1.7	4338.3 ± 1149.7
	14	49.6 ± 2.6	8.3 ± 1.5	42.2 ± 5.1	2481.7 ± 526.9
	21	76.6 ± 4.3	7.2 ± 0.8	16.1 ± 1.5	2140.0 ± 649.6

## Data Availability

All data used to support the findings of this study are available from the corresponding author upon request.

## Supplementary Materials

The following supporting information can be downloaded at: https://www.mdpi.com/article/10.3390/cells12101427/s1, Figure S1: Increased THP-1 initial cell seeding density increased the number of THP-1 macrophages (THP-1m) in a 5-day culture experiment. (A) Microscopic images showing cell morphology. (B) Percentage of cell confluency of THP-1m. (C) Percentage of detached cells. (D) Representative image of crystal violet staining on day 5, classified by cell number. (E) Quantitative results of crystal violet staining on day 5. (F) Metabolic activity rate of THP-1m by resazurin assay. Values are presented as mean ± standard deviation. * Statistically significant (*p* < 0.05); Figure S2: Long-term culture of THP-1m in RPMI-1640 and DMEM. (A) Live images of cell morphology. (B) Whole-well images of crystal violet staining on day 21; Figure S3: Hoechst staining of nuclei of THP-1 monocyte cells. Arrowheads indicate THP-1 monocytes that cannot be stained with Hoechst dye; Figure S4: Mucus staining with Alcian blue of the Caco-2, HT-29, and HT-29-MTX monolayer and the Caco-2/HT-29-MTX co-culture at a ratio of 9:1 after 21 days of culture; Table S1: Morphological parameters and size of the nucleus prepared in THP-1 monocytes and THP-1m. Data are presented as average ± standard deviation (n = 89).

## References

[1] Blom R.A., Erni S.T., Krempaská K., Schaerer O., Van Dijk R.M., Amacker M., Moser C., Hall S.R., Von Garnier C., Blank F. (2016). A triple co-culture model of the human respiratory tract to study immune-modulatory effects of liposomes and virosomes. PLoS ONE.

[2] Kämpfer A.A., Urbán P., Gioria S., Kanase N., Stone V., Kinsner-Ovaskainen A. (2017). Development of an in vitro co-culture model to mimic the human intestine in healthy and diseased state. Toxicol. In Vitro.

[3] Fedi A., Vitale C., Ponschin G., Ayehunie S., Fato M., Scaglione S. (2021). In vitro models replicating the human intestinal epithelium for absorption and metabolism studies: A systematic review. J. Control. Release.

[4] Al-Ghadban S., Kaissi S., Homaidan F.R., Naim H.Y., El-Sabban M.E. (2016). Cross-talk between intestinal epithelial cells and immune cells in inflammatory bowel disease. Sci. Rep..

[5] Satsu H., Ishimoto Y., Nakano T., Mochizuki T., Iwanaga T., Shimizu M. (2006). Induction by activated macrophage-like THP-1 cells of apoptotic and necrotic cell death in intestinal epithelial Caco-2 monolayers via tumor necrosis factor-alpha. Exp. Cell Res..

[6] Stephens M., von der Weid P.-Y. (2020). Lipopolysaccharides modulate intestinal epithelial permeability and inflammation in a species-specific manner. Gut Microb..

[7] Duweb A., Gaiser A.K., Stiltz I., El Gaafary M., Simmet T., Syrovets T. (2022). The SC cell line as an in vitro model of human monocytes. J. Leukoc. Biol..

[8] Chanput W., Mes J.J., Wichers H.J. (2014). THP-1 cell line: An in vitro cell model for immune modulation approach. Int. Immunopharmacol..

[9] Fujiwara N., Kobayashi K. (2005). Macrophages in inflammation. Curr. Drug Targets-Inflamm. Allergy.

[10] Kämpfer A.A., Urbán P., La Spina R., Jiménez I.O., Kanase N., Stone V., Kinsner-Ovaskainen A. (2020). Ongoing inflammation enhances the toxicity of engineered nanomaterials: Application of an in vitro co-culture model of the healthy and inflamed intestine. Toxicol. In Vitro.

[11] Marescotti D., Lo Sasso G., Guerrera D., Renggli K., Ruiz Castro P.A., Piault R., Jaquet V., Moine F., Luettich K., Frentzel S. (2021). Development of an Advanced Multicellular Intestinal Model for Assessing Immunomodulatory Properties of Anti-Inflammatory Compounds. Front. Pharmacol..

[12] Moyes S.M., Morris J.F., Carr K.E. (2010). Macrophages increase microparticle uptake by enterocyte-like Caco-2 cell monolayers. J. Anat..

[13] Pinto S.M., Kim H., Subbannayya Y., Giambelluca M., Bösl K., Kandasamy R.K. (2020). Dose-dependent phorbol 12-myristate-13-acetate-mediated monocyte-to-macrophage differentiation induces unique proteomic signatures in THP-1 cells. bioRxiv.

[14] Lund M.E., To J., O’Brien B.A., Donnelly S. (2016). The choice of phorbol 12-myristate 13-acetate differentiation protocol influences the response of THP-1 macrophages to a pro-inflammatory stimulus. J. Immunol. Methods.

[15] Ota T., Jiang Y.S., Fujiwara M., Tatsuka M. (2017). Apoptosis-independent cleavage of RhoGDIβ at Asp19 during PMA-stimulated differentiation of THP-1 cells to macrophages. Mol. Med. Rep..

[16] Kletting S., Barthold S., Repnik U., Griffiths G., Loretz B., Schneider-Daum N., de Souza Carvalho-Wodarz C., Lehr C.-M. (2018). Co-culture of human alveolar epithelial (hAELVi) and macrophage (THP-1) cell lines. ALTEX Altern. Anim. Exp..

[17] Aldo P.B., Craveiro V., Guller S., Mor G. (2013). Effect of Culture Conditions on the Phenotype of THP-1 Monocyte Cell Line.

[18] Sureshbabu A., Okajima H., Yamanaka D., Tonner E., Shastri S., Maycock J., Szymanowska M., Shand J., Takahashi S.-I., Beattie J. (2012). IGFBP5 induces cell adhesion, increases cell survival and inhibits cell migration in MCF-7 human breast cancer cells. J. Cell Sci..

[19] Jakic B., Buszko M., Cappellano G., Wick G. (2017). Elevated sodium leads to the increased expression of HSP60 and induces apoptosis in HUVECs. PLoS ONE.

[20] Grytting V.S., Olderbø B.P., Holme J.A., Samuelsen J.T., Solhaug A., Becher R., Bølling A.K. (2019). Di-n-butyl phthalate modifies PMA-induced macrophage differentiation of THP-1 monocytes via PPARγ. Toxicol. In Vitro.

[21] Lesuffleur T., Barbat A., Dussaulx E., Zweibaum A. (1990). Growth adaptation to methotrexate of HT-29 human colon carcinoma cells is associated with their ability to differentiate into columnar absorptive and mucus-secreting cells. Cancer Res..

[22] Schimpel C., Teubl B., Absenger M., Meindl C., Fröhlich E., Leitinger G., Zimmer A., Roblegg E. (2014). Development of an advanced intestinal in vitro triple culture permeability model to study transport of nanoparticles. Mol. Pharm..

[23] Srinivasan B., Kolli A.R., Esch M.B., Abaci H.E., Shuler M.L., Hickman J.J. (2015). TEER measurement techniques for in vitro barrier model systems. SLAS Technol..

[24] Kayama H., Okumura R., Takeda K. (2020). Interaction between the microbiota, epithelia, and immune cells in the intestine. Ann. Rev. Immunol..

[25] Oishi Y., Manabe I. (2018). Macrophages in inflammation, repair and regeneration. Int. Immunol..

[26] Watanabe S., Alexander M., Misharin A.V., Budinger G.S. (2019). The role of macrophages in the resolution of inflammation. J. Clin. Investig..

[27] Austermann J., Roth J., Barczyk-Kahlert K. (2022). The good and the bad: Monocytes’ and macrophages’ diverse functions in inflammation. Cells.

[28] Gordon S., Martinez-Pomares L. (2017). Physiological roles of macrophages. Pflügers Arch. Eur. J. Physiol..

[29] Daigneault M., Preston J.A., Marriott H.M., Whyte M.K., Dockrell D.H. (2010). The identification of markers of macrophage differentiation in PMA-stimulated THP-1 cells and monocyte-derived macrophages. PLoS ONE.

[30] Madhvi A., Mishra H., Leisching G., Mahlobo P., Baker B. (2019). Comparison of human monocyte derived macrophages and THP1-like macrophages as in vitro models for M. tuberculosis infection. Comp. Immunol. Microbiol. Infect. Dis..

[31] Jimenez-Duran G., Luque-Martin R., Patel M., Koppe E., Bernard S., Sharp C., Buchan N., Rea C., de Winther M.P., Turan N. (2020). Pharmacological validation of targets regulating CD14 during macrophage differentiation. EBioMedicine.

[32] Li Z.H., Si Y., Xu G., Chen X.M., Xiong H., Lai L., Zheng Y.Q., Zhang Z.G. (2017). High-dose PMA with RANKL and MCSF induces THP-1 cell differentiation into human functional osteoclasts in vitro. Mol. Med. Rep..

[33] Park E.K., Jung H.S., Yang H.I., Yoo M.C., Kim C., Kim K.S. (2007). Optimized THP-1 differentiation is required for the detection of responses to weak stimuli. Inflammat. Res..

[34] Starr T., Bauler T.J., Malik-Kale P., Steele-Mortimer O. (2018). The phorbol 12-myristate-13-acetate differentiation protocol is critical to the interaction of THP-1 macrophages with Salmonella Typhimurium. PLoS ONE.

[35] Wang L., Zhu L., Duan C., Li L., Chen G. (2020). Total saponin of Dioscorea collettii attenuates MSU crystal-induced inflammation via inhibiting the activation of the NALP3 inflammasome and caspase-1 in THP-1 macrophages. Mol. Med. Rep..

[36] Wang Y., Yin C., Feng L., Ma L., Wei Y., Sheng G. (2013). Sorting, identification and enrichment of side population cells in THP-1 acute monocytic leukemia cells. Oncol. Rep..

[37] Shahar M., Szalat A., Rosen H. (2021). Pathogenic Stress Induces Human Monocyte to Express an Extracellular Web of Tunneling Nanotubes. Front. Immunol..

[38] Sheng L., Leshchyns’ka I., Sytnyk V. (2013). Cell adhesion and intracellular calcium signaling in neurons. Cell Commun. Signal..

[39] Smith B.M., Sturm R.J., Carchman R.A. (1983). Calcium modulation of phorbol ester-induced alterations in murine macrophage morphology. Cancer Res..

[40] Chauhan A., Sun Y., Sukumaran P., Zangbede F.O.Q., Jondle C.N., Sharma A., Evans D.L., Chauhan P., Szlabick R.E., Aaland M.O. (2018). M1 macrophage polarization is dependent on TRPC1-mediated calcium entry. iScience.

[41] Długosz E., Basałaj K., Zawistowska-Deniziak A. (2019). Cytokine production and signalling in human THP-1 macrophages is dependent on Toxocara canis glycans. Parasitol. Res..

[42] Serrano C., Galán S., Rubio J.F., Candelario-Martínez A., Montes-Gómez A.E., Chánez-Paredes S., Cedillo-Barrón L., Schnoor M., Meraz-Ríos M.A., Villegas-Sepúlveda N. (2019). Compartmentalized response of IL-6/STAT3 signaling in the colonic mucosa mediates colitis development. J. Immunol..

[43] Skronska-Wasek W., Durlanik S., Garnett J., Pflanz S. (2021). Polarized cytokine release from airway epithelium differentially influences macrophage phenotype. Mol. Immunol..

[44] Armstead A.L., Li B. (2016). In vitro inflammatory effects of hard metal (WC–Co) nanoparticle exposure. Int. J. Nanomed..

[45] Segura M., Vadeboncoeur N., Gottschalk M. (2002). CD14-dependent and-independent cytokine and chemokine production by human THP-1 monocytes stimulated by Streptococcus suis capsular type 2. Clin. Exp. Immunol..

[46] Groeneweg M., Kanters E., Vergouwe M.N., Duerink H., Kraal G., Hofker M.H., de Winther M.P. (2006). Lipopolysaccharide-induced gene expression in murine macrophages is enhanced by prior exposure to oxLDL. J. Lipid Res..

[47] Calatayud M., Dezutter O., Hernandez-Sanabria E., Hidalgo-Martinez S., Meysman F.J., Van de Wiele T. (2019). Development of a host-microbiome model of the small intestine. FASEB J..

[48] Lehner R., Wohlleben W., Septiadi D., Landsiedel R., Petri-Fink A., Rothen-Rutishauser B. (2020). A novel 3D intestine barrier model to study the immune response upon exposure to microplastics. Arch. Toxicol..

[49] Gibb M., Pradhan S.H., Mulenos M.R., Lujan H., Liu J., Ede J.D., Shatkin J.A., Sayes C.M. (2021). Characterization of a human in vitro intestinal model for the hazard assessment of nanomaterials used in cancer immunotherapy. Appl. Sci..

[50] Weber L., Kuck K., Jürgenliemk G., Heilmann J., Lipowicz B., Vissiennon C. (2020). Anti-inflammatory and barrier-stabilising effects of myrrh, coffee charcoal and chamomile flower extract in a co-culture cell model of the intestinal mucosa. Biomolecules.

[51] Ling X., Linglong P., Weixia D., Hong W. (2016). Protective effects of bifidobacterium on intestinal barrier function in LPS-induced enterocyte barrier injury of Caco-2 monolayers and in a rat NEC model. PLoS ONE.

[52] Tu J., Xu Y., Xu J., Ling Y., Cai Y. (2016). Chitosan nanoparticles reduce LPS-induced inflammatory reaction via inhibition of NF-κB pathway in Caco-2 cells. Int. J. Biol. Macromol..

[53] Felix K., Tobias S., Jan H., Nicolas S., Michael M. (2021). Measurements of transepithelial electrical resistance (TEER) are affected by junctional length in immature epithelial monolayers. Histochem. Cell Biol..

[54] Hoffmann P., Burmester M., Langeheine M., Brehm R., Empl M.T., Seeger B., Breves G. (2021). Caco-2/HT29-MTX co-cultured cells as a model for studying physiological properties and toxin-induced effects on intestinal cells. PLoS ONE.

[55] Hasbullah N.I., Syed Mohamad S.A., Hasan N.A., Ahmad N., Johari N.A., Abd Manap M.N., Mohd Amin M.C.I. (2022). The role of mucus in adhesion and invasion of foodborne pathogens: Challenges in current human intestinal model. Food Res..

[56] Hu W., Feng P., Zhang M., Tian T., Wang S., Zhao B., Li Y., Wang S., Wu C. (2022). Endotoxins Induced ECM-Receptor Interaction Pathway Signal Effect on the Function of MUC2 in Caco2/HT29 Co-Culture Cells. Front. Immunol..

[57] Guo S., Al-Sadi R., Said H.M., Ma T.Y. (2013). Lipopolysaccharide causes an increase in intestinal tight junction permeability in vitro and in vivo by inducing enterocyte membrane expression and localization of TLR-4 and CD14. Am. J. Pathol..

[58] Busch M., Bredeck G., Kämpfer A.A., Schins R.P. (2021). Investigations of acute effects of polystyrene and polyvinyl chloride micro-and nanoplastics in an advanced in vitro triple culture model of the healthy and inflamed intestine. Environ. Res..

[59] Mowat A.M., Agace W.W. (2014). Regional specialization within the intestinal immune system. Nat. Rev. Immunol..

[60] Wang S., Ye Q., Zeng X., Qiao S. (2019). Functions of macrophages in the maintenance of intestinal homeostasis. J. Immunol. Res..

[61] Kaulmann A., Legay S., Schneider Y.J., Hoffmann L., Bohn T. (2016). Inflammation related responses of intestinal cells to plum and cabbage digesta with differential carotenoid and polyphenol profiles following simulated gastrointestinal digestion. Mol. Nutrit. Food Res..

[62] Ponce de León-Rodríguez M.d.C., Guyot J.-P., Laurent-Babot C. (2019). Intestinal in vitro cell culture models and their potential to study the effect of food components on intestinal inflammation. Crit. Rev. Food Sci. Nutrit..

[63] Costa J., Ahluwalia A. (2019). Advances and current challenges in intestinal in vitro model engineering: A digest. Front. Bioeng. Biotechnol..

[64] Kakni P., Truckenmüller R., Habibović P., van Griensven M., Giselbrecht S. (2022). A Microwell-Based Intestinal Organoid-Macrophage Co-Culture System to Study Intestinal Inflammation. Int. J. Mol. Sci..

[65] Bi Y., Shirure V.S., Liu R., Cunningham C., Ding L., Meacham J.M., Goedegebuure S.P., George S.C., Fields R.C. (2020). Tumor-on-a-chip platform to interrogate the role of macrophages in tumor progression. Integrat. Biol..

[66] Prestigiacomo V., Weston A., Messner S., Lampart F., Suter-Dick L. (2017). Pro-fibrotic compounds induce stellate cell activation, ECM-remodelling and Nrf2 activation in a human 3D-multicellular model of liver fibrosis. PLoS ONE.

[67] Sasserath T., Rumsey J.W., McAleer C.W., Bridges L.R., Long C.J., Elbrecht D., Schuler F., Roth A., Bertinetti-LaPatki C., Shuler M.L. (2020). Differential monocyte actuation in a three-organ functional innate immune system-on-a-chip. Adv. Sci..

