# Supplementary material for: Optimizing THP-1 Macrophage Culture for an Immune-Responsive Human Intestinal Model
Source: Cells. 2023 May 19;12(10):1427. doi: 10.3390/cells12101427 (PMC10217152; doi:10.3390/cells12101427)
Supplement: Supplementary file 1 [file cells-12-01427-s001.zip › cells-2305670-supplementary.pdf]

Supplementary Data

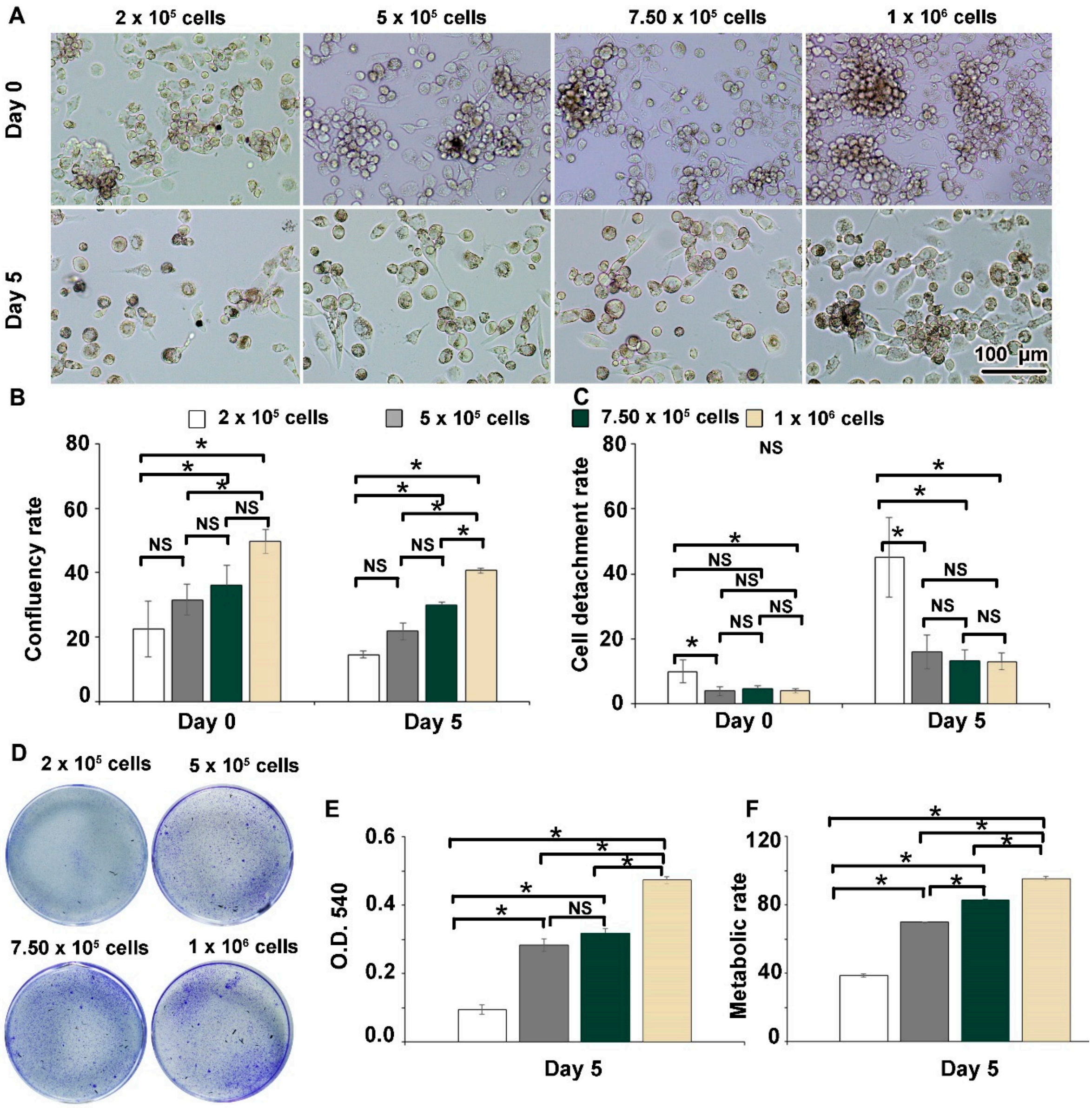

Figure S1. Increased THP-1 initial cell seeding density increased the number of THP-1 macrophages (THP-1m) in a 5-day culture experiment in RPMI-1640. (**A**) Microscopic images showing cell morphology. (**B**) Percentage of cell confluency of THP-1m. (**C**) Percentage of detached cells (**D**) Representative image of crystal violet staining on day 5, classified by cell number. (**E**) Quantitative results of crystal violet staining on day 5. (**F**) Metabolic activity rate of THP-1m by resazurin assay. Values are presented as mean ± standard deviation. *Statistically significant ($p < 0.05$).

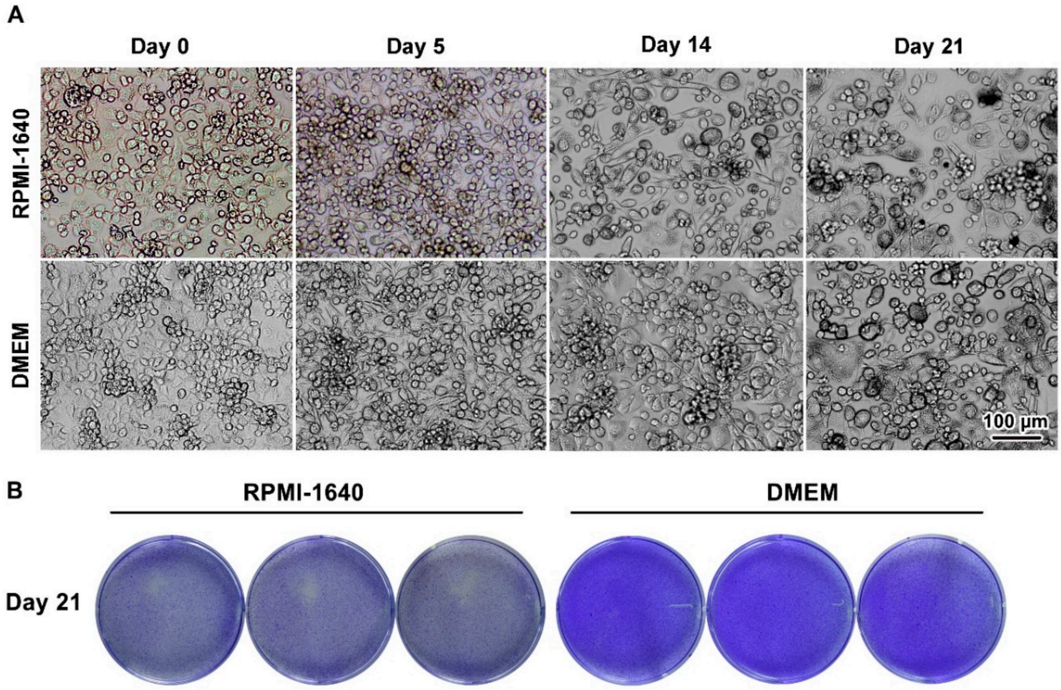

Figure S2. Long-term culture of THP-1m in RPMI-1640 and DMEM. (**A**) Live images of cell morphology. (**B**) Whole-well images of crystal violet staining on day 21.

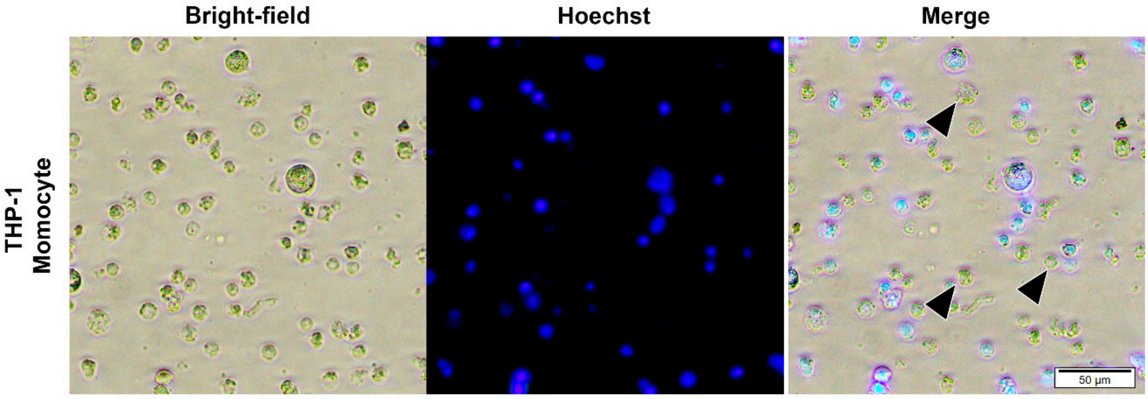

Figure S3. Hoechst staining of nuclei of THP-1 monocyte cells. Arrowheads indicate THP-1 monocytes that cannot be stained with Hoechst dye.

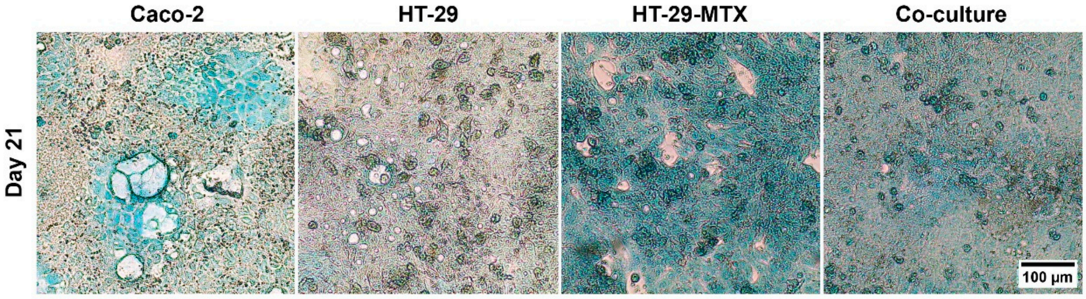

Figure S4. Mucus staining with Alcian blue of the Caco-2, HT-29, and HT-29-MTX monolayer and the Caco-2/HT-29-MTX co-culture at a ratio of 9:1 after 21 days of culture.

Table S1. Morphological parameters and size of the nucleus prepared in THP-1 monocytes and THP-1m. Data are presented as average ± standard deviation (n = 89).

**Type**	**Mean ±S.D.**			
	**Max Feret (μm)**	**Min Feret (μm)**	**Ratio**	**Nucleus Size (μm)**
THP-1 monocyte	11.3±1.6	10.0±1.5	1.1±0.1	10.6±1.5
Round	17.4±4.0	14.7±3.7	1.2±0.1	24.8±1.5
Fried egg	33.1±6.2	27.8±5.1	1.2±0.1	46.8±8.7
Spindle-like	28.4±5.1	21.9±5.1	1.3±0.1	39.4±7.0
